# Breast Tumour Classification Using Ultrasound Elastography with Machine Learning: A Systematic Scoping Review

**DOI:** 10.3390/cancers14020367

**Published:** 2022-01-12

**Authors:** Ye-Jiao Mao, Hyo-Jung Lim, Ming Ni, Wai-Hin Yan, Duo Wai-Chi Wong, James Chung-Wai Cheung

**Affiliations:** 1 Department of Bioengineering, Imperial College, London SW7 2AZ, UK; yejiao.mao20@imperial.ac.uk; 2 Department of Biomedical Engineering, Faculty of Engineering, The Hong Kong Polytechnic University, Hong Kong 999077, China; hyo-jung.lim@connect.polyu.hk; 3 School of Health Science and Engineering, University of Shanghai for Science and Technology, Shanghai 200093, China; gendianqing@163.com; 4 Department of Orthopaedics, Pudong New Area People’s Hospital Affiliated to Shanghai University of Medicine and Health Science, Shanghai 201299, China; 5 Department of Economics, The Chinese University of Hong Kong, Hong Kong 999077, China; whyan@cuhk.edu.hk; 6 Research Institute of Smart Ageing, The Hong Kong Polytechnic University, Hong Kong 999077, China

**Keywords:** breast cancer, breast neoplasm, benign, malignancy, computer-aided diagnosis, deep learning, artificial intelligence, CNN, shear wave elastography, sonoelastography

## Abstract

**Simple Summary:**

Breast cancer is one of the most common cancers among women globally. Early and accurate screening of breast tumours can improve survival. Ultrasound elastography is a non-invasive and non-ionizing imaging approach to characterize lesions for breast cancer screening, while machine learning techniques could improve the accuracy and reliability of computer-aided diagnosis. This review focuses on the state-of-the-art development and application of the machine learning model in breast tumour classification.

**Abstract:**

Ultrasound elastography can quantify stiffness distribution of tissue lesions and complements conventional B-mode ultrasound for breast cancer screening. Recently, the development of computer-aided diagnosis has improved the reliability of the system, whilst the inception of machine learning, such as deep learning, has further extended its power by facilitating automated segmentation and tumour classification. The objective of this review was to summarize application of the machine learning model to ultrasound elastography systems for breast tumour classification. Review databases included PubMed, Web of Science, CINAHL, and EMBASE. Thirteen (*n* = 13) articles were eligible for review. Shear-wave elastography was investigated in six articles, whereas seven studies focused on strain elastography (5 freehand and 2 Acoustic Radiation Force). Traditional computer vision workflow was common in strain elastography with separated image segmentation, feature extraction, and classifier functions using different algorithm-based methods, neural networks or support vector machines (SVM). Shear-wave elastography often adopts the deep learning model, convolutional neural network (CNN), that integrates functional tasks. All of the reviewed articles achieved sensitivity ≥80%, while only half of them attained acceptable specificity ≥95%. Deep learning models did not necessarily perform better than traditional computer vision workflow. Nevertheless, there were inconsistencies and insufficiencies in reporting and calculation, such as the testing dataset, cross-validation, and methods to avoid overfitting. Most of the studies did not report loss or hyperparameters. Future studies may consider using the deep network with an attention layer to locate the targeted object automatically and online training to facilitate efficient re-training for sequential data.

## 1. Introduction

Breast cancer is the leading cause of death with the second-highest mortality rate among cancers affecting women [1,2,3]. Breast cancer has surpassed liver cancer and become the fourth most commonly diagnosed cancer, with new cases increasing from 0.3 million in 2015 to 0.42 million in 2020 [4]. It is also ranked with the highest incidence rate for cancer [4]. There is one breast cancer patient in every four cancer cases in females, while breast cancer accounts for one in six cancer deaths [5]. The financial burden of breast cancer is enormous. Women with breast cancer spend $13,000 more for healthcare expenses annually than those without breast cancer. In the United States, the cost of breast cancer screening exceeded USD 1 billion annually in 2006 [6] but was believed to be cost-effective to improve health benefits and reduce deaths [7]. Accurate screening and early diagnosis could lead to early and effective prevention and could be why developed countries have a higher survival rates than developing countries [1,3,8].

While breast self-examination using manual palpation is promoted, clinical mammograms remain the primary modality for asymptomatic breast cancer screening that is proven to be clinically evident and able to reduce the mortality rate [9,10]. However, ionizing radiation of mammograms may add carcinogenic risks and has been blamed for frequent overdiagnosis [11,12]. Besides that, breast magnetic resonance imaging (MRI) is used to diagnose primary malignancy and perform preoperative evaluations with high accuracy [13,14]. However, both mammograms and breast MRI are confined to the hospital setting and may not be suitable for large cohort screening because of their high cost and complicated operation [15]. This is of particular concern to developing countries with limited healthcare resources but higher breast cancer mortality [3,8,16].

Real-time B-mode ultrasound has emerged as an alternative imaging technique despite the fact that small tumours could be challenging to identify and occluded by the sternum and ribs [17]. In addition, speckle noise and low contrast in B-mode may impede the observation features to identify potential abnormalities. With the integration of another ultrasound imaging approach, ultrasound elastography can measure and quantify the stiffness distribution or differences of the soft tissue for tumour detection, under the premise that the lesion of breast tumours exhibits higher shear elasticity [18]. Ultrasound elastography was pioneered by Ophir et al. [19] in 1991. This elasticity imaging technique complements conventional B-mode imaging by superimposing stiffness measures onto the spatial information. Radiologists could conduct the assessment or diagnosis based on the Breast Imaging Reporting and Data System (BIRADS) protocol [20]. With the development of the extended combined autocorrelation method for lesion tracking, real-time freehand strain elastography could demonstrate good diagnostic performance in differentiating benign and malignant lesions [21]. Later, real-time shear wave elastography was invented in an attempt to remedy the problem of manual palpation [22], while some researchers further advanced the technique by incorporating colour Doppler into the shear wave imaging to improve the visualization of the shear wave wavefront [23]. Nowadays, ultrasound imaging with elastography has improved the sensitivity of small breast tumour detection [24], demonstrated high specificity for breast cancer diagnosis and become one of the prior examinations before the invasive breast biopsy [25].

There are still limitations with integrated B-mode and ultrasound elastography in breast tumour detection. The operation of ultrasound is highly dependent on the physicians’ experience [26]. Measurement errors due to inter and intra-observer variability in probe placement/orientation and annotation are undeniable [26,27]. Moreover, it could be difficult to distinguish the lesion boundary between the normal and tumour tissue and that between benign and malignant lesions. The accuracy of the malignancy scoring system could be jeopardized by necrosis and liquefaction in malignant lesions, or mechanization and calcification in benign lesions [28,29].

In light of the system weaknesses, computer-aided diagnosis (CAD) has been developed to improve the reliability of the system and is facilitated by the identification of critical image features by medical experts. The machine learning approach, such as deep learning, can improve the objectivity and reliability of identification and annotation of features, thus further extending the strength of CAD by enabling automated segmentation and thus staging for breast tumours [30,31]. To this end, the objective of this study was to review the methods and accuracy performance of state-of-the-art machine learning techniques used in ultrasound elastography for breast tumour classification and shed light on the improvement of CAD for early and accurate screening of breast cancer.

## 2. Materials and Methods

### 2.1. Search Strategy

A systematic literature search was performed to review diagnostic studies involving breast cancer screening or breast tumour detection using ultrasound elastography and machine learning techniques. The literature search was conducted according to the Preferred Reporting Items for Systematic Review and Meta-Analysis Protocols Extension for Scoping Reviews (PRISMA-ScR) guidelines [32]. The literature search was performed on databases including PubMed (title/abstract, journal articles, English), Web of Science (topic field, articles, English), CINAHL via EBSCOhost (default field) and EMBASE via OVID (topic field, English). Two authors (Y.-J.M. and D.W.-C.W.) conducted independent searches in November 2021. The first author (Y.-J.M.) conducted the screening on abstracts and full-text, which was checked by the corresponding author (D.W.-C.W.). Any disagreement was resolved by seeking consensus with the other corresponding author (J.C.-W.C).

The search was conducted using a combination of keywords related to breast cancer, ultrasound elastography and machine learning. For breast cancer, the search keywords included those with “breast” or “mammary” and those with “neoplasm*”, “tumo*r*”, “cancer”, “malignan*”, or “carcinoma*”. For search on PubMed, the search keywords were replaced by the MeSH term, “breast neoplasms”. For ultrasound elastography, the search keywords included “elastograph*”, “tissue stiffness”, or “modulus measure*”. For machine learning, the search keywords included “machine learning”, “deep learning”, “supervised learning”, “unsupervised learning”, “SVM”, “support vector machine”, “XGBoost”, “decision tree”, “optical flow”, “dynamic timewrap*”,”template match*”, “CNN”, “neural network”, “FCN”, “fully-connected network”, “fully connected network”, “Mask-RCNN”, “semantic segmentation”, “active contour”, “gradient vector flow”, “variation* auto-encoder”, “grabcut”, “adaptive thresholding”, “instance segmentation”, “threshold segmentation”, “edge detection segmentation”, or “mixture of Gaussian*”.

The search was limited to original journal research articles in English. The inclusion criteria included: (1) screening by both B-mode ultrasound and ultrasound elastography; (2) machine learning technique either in image segmentation, feature extraction, or classification; (3) diagnostic/screening accuracy test to classify benign and malignant breast tumours; (4) test involved with and evaluated by human subject data; (5) at least one accuracy performance measure. Studies were excluded if they: (1) targeted axillary lymph node breast cancer; (2) were non-machine learning techniques in all the three aforementioned aspects; (3) had insufficient details on the machine learning model; (4) involved additional modality other than B-mode ultrasound and elastography; (5) were modelled or evaluated by simulation data.

### 2.2. Screening and Data Extraction

**Figure 1:**
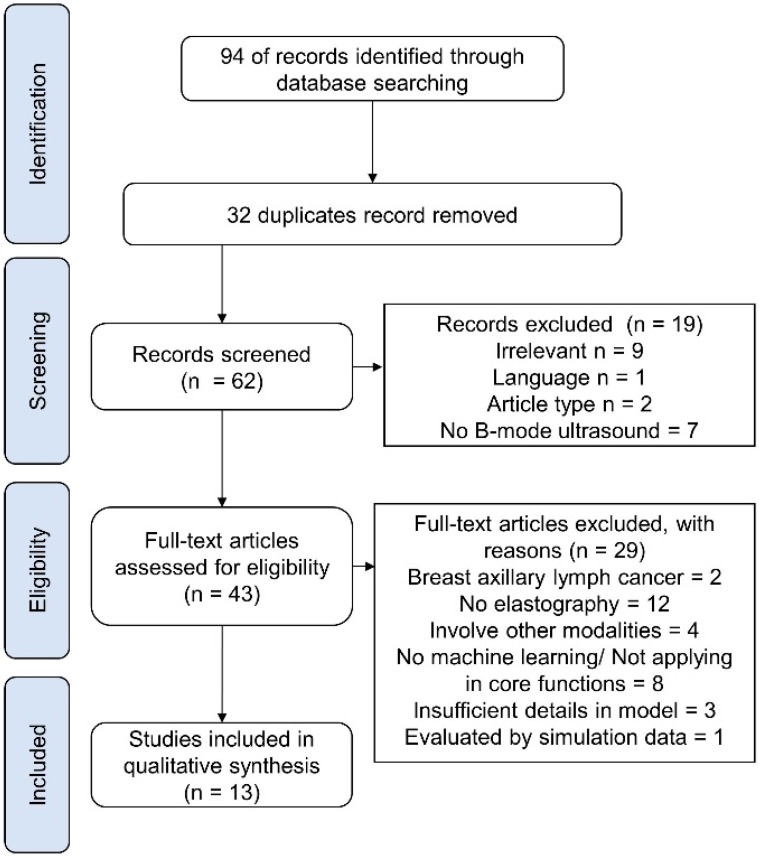
PRISMA flowchart of systematic search and selection process.

The search and screening process for the systematic review is shown in Figure 1. There was no disagreement among authors in the selection of studies for the review. The review context included basic information on subject information and dataset (Table 1), the configuration of the ultrasound system, image pre-processing and segmentation (Table 2), feature extraction, fusion, and reduction, classification (Table 3), evaluation metrics and performance (Table 4).

## 3. Results

### 3.1. Search Results

As shown in Figure 1, the initial search identified 94 articles. After the removal of duplicates, 62 articles were eligible for screening. A primary screening excluded 19 articles, with reasons (irrelevant, *n* = 9; language, *n* = 1; article type, *n* = 2; no B-mode ultrasound, *n* = 7). A full-text screening excluded 29 articles with reasons (breast axillary lymph cancer, *n* = 2; no elastography, *n* = 12; involved other modalities, *n* = 4; evaluated by simulation data = 1; no machine learning or not used on core functions, *n* = 8; insufficient details of the model, *n* =3). In the end, 13 articles were eligible for data synthesis [33,34,35,36,37,38,39,40,41,42,43,44,45].

### 3.2. Basic Information and Dataset

The 13 articles involved a total of 1988 participants with a dataset of 3216 tumour images (1708 benign and 1508 malignant), as shown in Table 1. The sample size for patients ranged from 80 to 363, while all of them had at least 100 image samples. It should be noted that articles coming from the same research team were likely to have the same set of participants or source data based on the demographic information—for example, the articles among research teams of Sasikala et al. [37,38], Wu et al. [39,40], and Zhang et al. [42,43]. There was also a mismatch between the sample size of patients and dataset images, which could be due to multiple lesions from the same patient justified by a few articles. Based on the available data, the age range was from 16 to 97. Most articles (11 out of 13) indicated that diagnosis (reference standard, or ground truth) of benign or malignant lesion was made by biopsy or histopathology. Among them, three of the articles noted that biopsy tests were conducted only for those screened by ultrasound or other modes of examination. The lesion size information of seven articles was not available and could be an influencing factor towards classification performance.

An equal number of studies collected data retrospectively or prospectively (*n* = 6), while one study did not present the respective details [33]. Four studies highlighted the proportion of data used for model training and independent testing, which was approximately 75% to 80% for model training [34,35,44,45]. Two of them involved an additional dataset for external testing [44,45], and one dataset was sourced from a hospital different from the model training dataset [44]. Five studies neither addressed the division of model training and testing dataset, nor described a cross-validation, while two studies used a cross-validation [42,43]. Cross-validation directs different proportions of data for training and testing on different iterations [46]. For example, a 5-fold cross-validation splits the dataset into 5 proportions of equal size (fold). Four folds are used to train the model, and one fold is used for testing, in which the process is repeated for each fold. Similarly, the leave-one-out cross-validation picks one sample for testing and repeats the process until all samples are exhausted. Essentially, the performance evaluation would be computed by the average performance of the iterations. Nevertheless, nearly half (*n* = 6) of the studies applied the cross-validation, as shown in Table 3.

To “enlarge” the sample size for model training, the data augmentation technique is often used in the field of machine learning to facilitate convergence and robustness. As shown in Table 3, five studies implemented the data augmentation procedure [34,35,44,45]. The classic data augmentation technique involves image flipping, random rotation, and rescaling.

## 4. Review Theme and Context

### 4.1. Ultrasound Elastography

Out of the 13 articles, six applied shear wave elastography (SWE). In contrast, the others involved strain elastography (SE) using freehand (FH)/an externally applied force (*n* = 5) or acoustic radiation force (ARF) (*n* = 2), as shown in Table 2.

SE estimates elastic modulus by the ratio of known force over a compression area to the ultrasound-measured dimension depth change of the soft tissue (strain) [47]. The system targets lesions near the surface at about 5 cm depth [48]. The advantage is that it is convenient for real-time strain visualization [48]. However, the externally applied compression is conducted freehand, in which the data collection quality may be dependent on the operators’ experience and subject to interobserver variability [49]. The semiquantitative compensation of this problem by B-mode ultrasound may hinder estimation of the exact elasticity values [50,51]. Some other researchers attempted to generate three-dimensional elastography by SE images [52]. ARF on SE remedies this problem by a controlled pushing pulse to induce tissue displacement, which is followed by an ultrasound pulse to capture the position and displacement of the tissue. It is more effective than freehand SE in measuring deeper tissues [48].

SWE induces and measures the propagation speed of the shear wave (*c*), which is dependent on the density (*ρ*) and elastic modulus (*E*) of the tissue, *E = 3ρc*^2^ [48,53]. The strength of SWE is its reproducibility and the mapping of tissue elasticity onto the morphological information of the B-mode ultrasound, which improves the specificity of B-mode ultrasound without losing sensitivity [54,55], despite a higher cost. Stiffer non-homogeneous masses are more susceptible to malignancy [54]. Therefore, examining the peritumoral region could be more important than the lesion region itself [56].

### 4.2. Image Pre-Processing, and Segmentation

Image pre-processing techniques could involve cropping, resampling, denoising, conversion, and image separation, while some studies only lightly described in their routine procedures. Among the studies, Misra et al. [35] decided to compare the model performance with and without image cropping. Zhang et al. [42,43] and Zhou et al. [45] isolated and extracted the pure shear wave elastography for analysis by a technique (image separation) that subtracted the B-mode grayscale image from the composite colour image data and then calibrated the elasticity modulus [57,58]. Wu and colleagues attempted two different pre-processing techniques (Harris corner operation and fractional order operation) in two publications [39,40]. The fractional order operation method adopted a multiscale image approach to enhance the higher frequency components of the images (i.e., edge information) [59], while the Harris corner operation implemented the filter through convolution with a structured tensor [60].

For image segmentation, there could be manual segmentation, algorithm-based segmentation, deep learning models (bypassing image segmentation), or a mixture of the methods above. Moon et al. [36] conducted the manual segmentation for the region-of-interest (ROI) by radiologists without any pre-processing technique. Two papers involved manual segmentation after different pre-processing techniques [39,40]. Another article implemented manual segmentation and algorithm-based segmentation together [41]. Level sets and fuzzy level sets were algorithm-based methods that used a threshold or a fuzzy-threshold level segmentation and were applied in five articles. 

Sometimes, image pre-processing and segmentation procedures were indistinguishable because some pre-processing techniques were essential steps to facilitate or reduce the burden for segmentation, such as image cropping and contouring. Anisotropic diffusion filtering with sticking, speckle reducing anisotropic diffusion (SRAD), Gabor-based anisotropic diffusion (GAD), and active contour were the common processes to remove speckle noise using an edge-sensitive technique computed by the function of local gradient or entropy magnitude [61], while it was also regarded as an image segmentation procedure.

Additionally, Zhang et al. [43] merged the GAD with reaction diffusion (RD) based level set segmentation. The significant contribution from Yu et al. [41] was that they proceeded with a series of pre-processing steps, including k-means clustering, active contour, and dyadic wavelet transform. The dyadic wavelet transform initialized the image into an energy field that could achieve a sufficient signal-to-noise ratio to drive the active contour, with the region then smoothened by GAD and refined by k-means clustering [41].

For the evaluation of image segmentation, some studies applied and evaluated the performance of manual segmentation [41,44]. Based on the spatial overlapping, the dice similarity coefficient was used to evaluate the intra and inter-rater reproducibility of segmentation [62], in addition to accuracy performance measures [43]. In contrast, some studies applied algorithm-based segmentation and evaluated by manual segmentation as the reference [41,43]. Chen et al. [33] believed that the detected edges of the segmented images were acceptable based on empirical verification by experienced radiologists. Distance-based measures, such as mean absolute distance, were used in two articles for evaluation [33,41].

### 4.3. Feature Extraction, Fusion, and Reduction

Generally, feature extraction and classification of the studies were based on two approaches or a mixture of these two approaches. The first approach was a deep learning workflow that embedded all relevant functions (image segmentation, feature extraction/reduction, classification) into the machine learning or deep learning model [63], particularly CNN. The second approach was to configure the feature extraction and classifier separately, also known as the traditional computer vision workflow [63].

For the feature extraction, three studies pre-determined the features to be used for classification [33,36,41], as shown in Table 3. Feature extraction techniques were generally based on the image presentation, such as pixel, intensity, grey level, etc. They included local binary pattern (LBP) [37,38], local ternary pattern (LTP) [37], grey level co-occurrence matrix (GLCM) [38], grey level difference method (GLDM) [38], LAWs texture energy measure [38], point-wise gated Boltzmann machine (PGBM) with restricted Boltzmann machine (RBM) [42], contourlet-based texture feature extraction [43], Harris corner convolution [39], and fractional order convolution [40]. On the one hand, a unique point of the contourlet-based texture feature extraction was that it integrated the tumour elasticity in the spatial-frequency domain with the morphological features for better classification [43]. On the other hand, PGBM utilized a gating mechanism using a stochastic switch unit to estimate whether the feature pattern occurred [42]. Besides, if the extracted features were radiomic parameters, least absolute shrinkage and selection operator (LASSO) regression could be applied to weigh selected features for reduction [44].

Feature fusion could also be implemented using serial fusion, parallel fusion, or particle swarm optimization (PSO). Instead of feature fusion, Wu and colleagues [39,40] applied the PSO model to improve model learning only, whilst Sasikala et al. [38] used an optimum path forest (OPF) to optimize the performance of PSO. Subsequently, the number of extracted features could be large, as many as 286, as demonstrated by Zhang et al. [42]. Feature reduction could be achieved by principal component analysis (PCA) [37], canonical correlation analysis (CCA) [37], deep polynomial network (DPN) [43], or multiple kernel learning (MKL) [43]. The advantage of the novel DPN was that it weighs and identifies high-level features over multiple output layers, which enables effective learning from small samples [43].

### 4.4. Classification

Support vector machine (SVM) was often used as the binary classifier with prior confirmed extracted features (*n* = 6), as shown in Table 3. SVM was recognized as the most robust and accurate classifier before deep learning [64]. It classified the data by a hyperplane with a dimensional space at the order of the number of features. Other classifiers included random decision forest [39], multilayer perceptron neural network (MPNN) [36], Bayesian classification [36], and generalized regression neural network (GRNN) [39,40].

### 4.5. Deep Learning

As mentioned in Section 4.3, the deep learning model, particularly CNN in this review, embedded all relevant functions (image segmentation, feature extraction/reduction, classification) and minimized any manual procedures or decision-making. The basic principle of CNN was to train a kernel (or filter) to recognize specific image features (convolution layer) [63]. The model then computed the level of feature overlapping between the kernel and the input image (known as the receptive field), followed by a pooling layer for higher-level features and a fully connected layer to flatten the data into a feature vector [65]. The output layer of the model computed the probability of the output class through a dense network and a regression function [66]. Fujioka et al. [34] and Misra et al. [35] embedded all relevant functions using a deep learning model, CNN. Before training the CNN, the authors pre-trained the model (or transfer learning) by ImageNet (https://www.image-net.org, accessed on 20 December 2021), which is a free image database organized according to WordNet Hierarchy [67], and has been recognized as the most commonly used dataset [68,69]. The transfer learning process trained the model by an existing large dataset before learning a specific scenario. Nevertheless, Fujioka et al. [34] and Misra et al. [35] sought different approaches in using CNN. Fujioka et al. [34] attempted and compared a pool of different CNN models, including Xception [70], InceptionV3 [71], InceptionNesNetV2 [72], DenseNet1 [73], DenseNet161 [74], and NASNetMobile [73]. In contrast, Misra et al. [35] selected two CNN models (AlexNet [75] and ResNet [76]) and integrated the models and ultrasound modalities (i.e., B-mode and SWE) by ensembled learning. On the other hand, Zhang et al. [44] and Zhou et al. [45] configurated the feature extraction and classifier separately, despite the application of CNN. A basic introduction to the different models is available in another scoping review [68].

### 4.6. Evaluation Metrics

**Figure 2:**
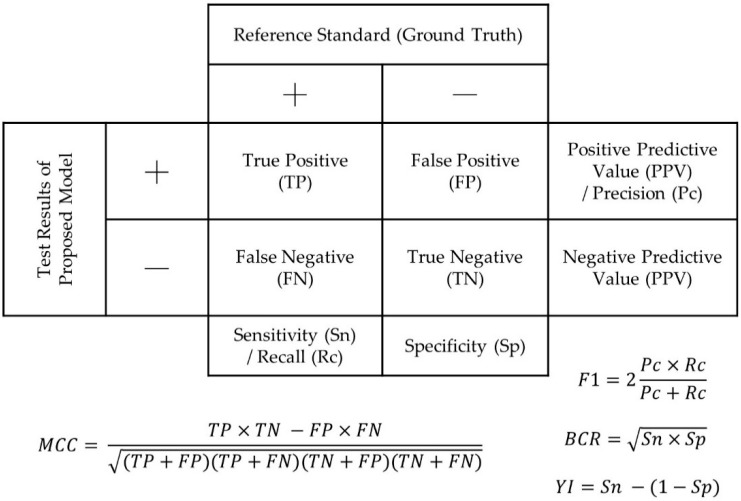
Confusion matrix demonstrating typical outcome measures used for model evaluation. (BCR/G-mean: balanced classification rate; MCC: Matthews correlation coefficient; YI: Youden’s index).

The evaluation metrics used in the articles were the same as the diagnostic metrics used in epidemiology, as shown in Figure 2. Sensitivity (or true positive rate) indicates the proportion of sample receiving a positive test result that actually has the condition, while specificity (or true negative rate) indicates the proportion of sample receiving a negative test result that actually does not have the condition. Positive predictive value (PPV) is the probability of having the condition with a positive test result, while the negative predictive value (NPV) is the probability of not having the condition with a negative test result. Accuracy is the fraction of correct test results over the total number of tests. However, the measure fails to account for the ratio between positive and negative tests and is thus not recommended to be used for highly imbalanced class problems that commonly appear in health science [77].

Recall and precision are two essential evaluation parameters in data science, which are equivalent to sensitivity and PPV. The different nomenclature is due to the concept of “relevance” in information retrieval. Recall indicates the percentage of relevant instances retrieved (recall), while precision is the fraction of relevant instances retrieved. The combination of recall and precision establishes some evaluation metrics. F1-score is the harmonic mean of recall and precision; balanced classification rate (BCR) is the geometric mean (G-mean) of recall and precision to avoid overfitting the negative class and underfitting the positive class [38]. The Matthews correlation coefficient (MCC) was proposed by Brian Matthews in 1975 [78] and was believed to be the most informative single metric for the evaluation of binary classifiers in prediction [79]. It quantifies the association between the ground truth and the prediction (test value) and is equivalent to the Phi coefficient in the Pearson chi-squared statistics.

The receiver-operating characteristics (ROC) curve is a standard tool to present the true positive rate as a function of false-positive rate for the continuum of all cut-off values for classification. The area under ROC curve (AUC) represents the probability of the classifier to correctly recognize the classes of a pair of randomly drawn positive and negative instances [80]. It serves as an overall performance indicator of discrimination capability, whilst Youden’s index (YI) evaluates the ability to avoid misclassification [35,37,38,39,40].

In biostatistics and epidemiology, the prediction or test is considered reliable with sensitivity ≥ 80%, specificity ≥ 95%, and PPV ≥ 95% [81,82]. As a rule of thumb, AUC ≥ 0.85 and 0.75 ≥ AUC ≥ 0.85 are considered convincing and partially convincing performance, respectively [83]. For machine learning or deep learning, we believe that accuracy or an F-score ≥ 90% is acceptable, while that ≥95% is good, with the premise that human labellers (ground truth) achieve 99% accuracy and the best model network achieves 95% accuracy on ImageNet [84].

### 4.7. Test Performance

The evaluation of models and systems in the articles often came with a comparison over different stages and aspects, which could be generally categorized into image pre-processing [34], image segmentation [37,39,40,42,43], feature extraction/reduction [37,38,39,40,41,42,43,44,45,46,47,48,49,50], and classifier/classifier settings [35,36,44,45]. Some of them compared multiple factors and levels. For example, Sasikala et al. [37] compared the performance between combinations of different image segmentation (LBP vs. LTP), feature fusion (serial vs. parallel), and reduction (PCA vs. CCA) techniques; Zhang et al. [42] compared the performance between combinations of different image segmentation (level set vs. PGBM vs. PGBM with RBM), feature reduction (PCA vs. t-test vs. no reduction), and classifier (ELM vs. KNN vs. SVM).

Table 4 highlights the results of either the proposed model or the best performing model in the articles. Nearly all articles applied sensitivity/recall and specificity as the primary outcome. Five studies used the F1-score to evaluate the model. Out of the 10 articles with available accuracy measures, the models of seven articles achieved an accuracy ≥ 90%. All models in the articles had a sensitivity ≥ 80%, while only half of them attained an acceptable specificity (i.e., ≥95%). However, it was interesting to know that cases that were tested wrong by the model were also misdiagnosed by radiologists [34]. All models with reported AUC (*n* = 6) demonstrated convincing classification performance. Deep learning models [34,35,44,45] did not necessarily perform better than the traditional computer vision approach.

Zhang et al. [44] reported a “perfect” test or model with 100% sensitivity and specificity and AUC = 1.0. It should be noted that the evaluation metric could be affected by overfitting when the model fits exactly against the training dataset. Cross-validation is a way to prevent overfitting [85], while some studies did not address how they handle overfitting or did not mention which dataset they used to calculate the evaluation metrics [33,34,36,37]. Moreover, the definition or calculation of evaluation metrics could be different, such as using cross-validation with different proportions [38,39,40,41,42,43] or testing datasets with different sample sizes [44,45]. Their findings may not be comparable, despite that some research was targeted to minimize manual operation rather than superior accuracy [41].

## 5. Remarks

Reporting quality is an essential component in the quality assessment of articles, including the investigations of machine learning [86]. More than half of the articles (9/13) clearly indicated the reference of the diagnosis (ground truth); nonetheless, a few (2/9) stated that the diagnostic test was only conducted for those screened positive and could be mistaken if the screening test had a low specificity. Out of the 13 articles, three specified neither the training and testing data set derivation nor cross-validation. One study applied an external testing set to improve generalizability [44]. Additionally, a few studies did not describe the demographic data (4/12) and lesion size (6/12), while two studies provided the details in the subgroups of training and testing set [34,44], and two studies in the subgroups of benign and malignant lesions [39,40]. Four studies included information relating to loss function or hyperparameters, though not all studies were applicable to those parameters. However, this information reflects how the training behavior of the model is controlled and has significant impact on model performance [87].

It should be noted that there were blatant examples of terminological confusion towards the training, testing, and validation dataset, while some studies were guilty of model peeking (i.e., the testing dataset was not completely separated from model training) [88]. The testing dataset should always be held out for the assessment of performance for the final tuned model only [89,90]. The training dataset is used for the model learning basically via fitting the parameters to the classifiers [89,90]. The validation dataset is used to optimize the model training by fine-tuning the hyperparameters and may serve as an intermediate evaluation. In the case of cross-validation (a bootstrap approach), the training, validation and testing datasets are nested without data splitting [91] and have been recommended for small sample sizes (e.g., <100), though this is controversial. Furthermore, Yusuf et al. [86] briefly noted that the nomenclature among communities is different. The validation set for a medical research community is equivalent to the testing set in the field of machine learning [86].

Segmentation-based methods could lead to the loss of peri-tumour and surrounding tissue information. The strain ratio between surrounding tissue and lesion is an important feature for classification and could not be calculated when the information of surrounding tissue is unknown. Moreover, inputting images without segmentation to the deep network demands higher computer resources and may lead to non-convergence or poor accuracy. Therefore, cropping an ROI at reasonable size to encompass the lesion and surrounding tissue is necessary. In fact, ultrasound has more difficultly in preserving peri-tumour tissue due to the limitations in image contrast, spatial resolution, and speckle noise. Pre-processing techniques, in particular smoothing, could overcome these limitations and are important to both automatic and manual segmentation. Nevertheless, the speckle information is a collection of echogenicity to reflect three-dimensional spatial information for surrounding issue, despite that the image is two-dimensional. Speckle literally contains morphological information of the surrounding tissues and has been used to estimate the motion of the ultrasound probe, such as the speckle decorrelation for three-dimensional reconstruction [92]. Moreover, the speckle “noise” could be extracted by the deep learning network as an important feature, while the smooth filter may weaken the irregular edge feature. Thus, it is controversial to completely smooth the image in the pre-processing stage.

We speculated an evolution of feature extraction techniques in deep learning, such that raw images are input instead of the smoothened and segmented images. It should also be noted that image compression may degrade the image quality and details, such as the use of JPEG [35]. A fuzzy level set method was used to accommodate the ambiguity and inhomogeneity of the image, which could be superior to the existing level set method [37,38]. We believe that the deep learning network could be more adaptive to noise during the image segmentation process.

In general, our review summarized that ultrasound elastography with machine learning was preceded either by traditional computer vision (traditional machine learning) or the deep learning approach. Traditional computer vision handled different functions of the workflow separately with different methods, such as manual or algorithm-based segmentation, and ended with a classifier, while the deep learning model, in particular CNN, integrated all the tasks [63]. Deep learning models are generally more reliable, time-consuming, and perform better than traditional algorithm-based methods or computer vision workflow. Instead of being programmed and using hand-crafted features, the deep learning models adopted an end-to-end learning approach that was trained with a class-annotated dataset to establish the most descriptive and salient features from the images [63]. For traditional computer vision, an expert in biomedical science, imaging, and computing is required to determine and justify the features to be extracted and the feature extraction methods, which could be a trial-and-error process requiring extensive time for fine-tuning and would be problematic in cases involving a plethora of features [63]. In addition, algorithms are more domain-specific, whereas models can always be trained by another dataset.

Traditional computer vision techniques are not without benefits. They are more computationally efficient and do not necessarily perform worse than deep learning models, as demonstrated in our review. Deep learning models require very demanding computer requirements and big datasets but lack explainability. The most common dataset, ImageNet, consists of over 1.5 million of images over thousands of object categories [93], though normally facilitated to the models by transfer learning. The lack of a large dataset may yield overfitting issues or reduce external validity that is often overlooked [94]. The full transparency in algorithm-based methods is also superior to the inscrutable Blackbox model to obtain physical meaning from the features and better insights into potential problems with the solutions, which could be imperative for clinicians [95]. The learning models would not only be confined to “garbage-in”, “garbage-out” [96], but also “garbage-learnt”.

There were some limitations in this review. First of all, the review was confined to journal articles written in English, which may lead to selection bias. In fact, many research articles in the fields of computing were published via conference full papers. Nevertheless, extensive efforts would be needed to screen conference materials for peer-reviewed full papers with sufficient context and quality. Secondly, we did not conduct a systematic analysis or meta-analysis for the diagnostic/screening performance in this review, though they had common evaluation metrics. There was high heterogeneity in the methods and dataset to generate the evaluation metrics among studies, such as cross-validation, external validation, or loss functions. Moreover, a number of studies did not account for over-fitting in their models that could overestimate the accuracy performance. A meta-analysis would likely mislead the readers during the comparison between systems and models. Furthermore, we confined the elastography review to strain or shear wave elastography, although the incorporation of ultrasound Doppler has received attention requiring development of specific machine learning techniques [23].

Attention layer [97] is increasingly applied in deep networks such as U-Net [98] to improve the performance of segmentation. It mimics the human cognitive attention function to focus on a particular object. A deep learning network with attention layer could guide the model to focus on a particular object in the image during the learning process. That approach can replace the segmentation process and improve the effectiveness of the learning and relevance of the extracted features. Currently, all input data are processed and pre-prepared before training. If there are new data, the model needs to be retrained for the full dataset. An online training method could be adopted, such that the model could be re-learnt and updated with sequential future data without retraining the whole dataset [99].

## Figures and Tables

**Table 1:** Subject information and dataset.

Article	Sample Size (Tn:Ts:Tx)	Mean Age (SD, Range)	Lesion Type (BT:MT)	Lesion Size (mm) (BT:MT)	Reference Standard (Diagnosis Modality)
Chen et al. [33]	86 patients 100 images	45 (-, 20–60)	60:40	-	Pathologically proven
Fujioka et al. [34]	363 patients 304: 73	Tn: 47.5 (13.1, 20–87) Ts: 47.7 (12.3, 30–82)	Tn: 158:146 Ts: 38:35	Tn (14.5:17.9) Ts (14.0:17.2)	-
Misra et al. [35]	85 patients 261 images 67:18	-	130: 131	-	Biopsy
Moon et al. [36]	171 patients	46 (-, 35–67)	101:39	10.1: 13.2	Ultrasound (BI-RADS), & some cases were biopsy
Sasikala et al. [37]	113 patients	-	62:51	-	-
Sasikala et al. [38]	113 patients	-	62:51	-	-
Wu et al. [39]	80 patients 320 images (1:1)	BT: 43.56 (11.34, 3–70) MT: 57.17 (12.7, 35–97)	34 (144 images): 46 (176 images)	40.67 (20.05): 38.65 (20.02)	Histopathology
Wu et al. [40]	80 patients 320 images (1:1)	BT: 43.56 (11.34, 31–70) MT: 57.17 (12.7, 35–97)	34 (144 images): 46 (176 images)	40.67 (20.05): 38.65 (20.02)	Histopathology
Yu et al. [41]	187 patients	41 (14, 16–77)	113: 74	-	Screened by B-mode then confirmed with biopsy
Zhang et al. [42]	121 patients (227 images)	39.9 (15.2, NS)	135: 92	0.54 (0.2) *	Biopsy
Zhang et al. [43]	121 patients (227 images)	-	135:92	-	Pathology
Zhang et al. [44]	263 patients 198:65:28	Tn: 40.47 (12.1, 18–77) Ts: 41.5 (13.2, 19–70)	Tn: 140:58 Ts: 46:19 Tx: 18:10	Tn: 13 (6, 4–34) Ts: 13 (6, 4–34)	Biopsy after mammogram, US & SWE examination
Zhou et al. [45]	205 patients 540 Images 400:45:95	35.6 (-, 16–79)	222:318	2–20	Biopsy

* Unit of the measurements was not available in the article. BT: benign tumours; BIRADS: Breast Imaging Reporting and Data System; MT: malignant tumours; SD: standard deviation; Tn: training set; Ts: testing set; Tx: external testing set/validation set; US: ultrasound; SWE: shear wave elastography.

**Table 2:** Configuration of the ultrasound system and image segmentation.

Article	Ultrasound System, Type		Image Pre-Processing	Image Segmentation	Evaluation of Segmentation
Chen et al. [33]	Voluson 530, Kretz Technik	SE-FH	Anisotropic diffusion filtering & stick technique	Level set method Subregion registration	Verified by Ro
Fujioka et al. [34]	Aplio 500, Toshiba	SWE	Manual cropping	CNN (Xception, Inception V3, InceptionNesNetV2, DenseNet1, DenseNet161, NASNetMobile) ^†^	-
Misra et al. [35]	Vision Ascendus, Hitachi	SE-FH	-	w/ vs. w/o manual cropping Ensembled CNN (AlexNet & ResNet) ^†^	-
Moon et al. [36]	EUB-8500, Hitachi	SE-FH	-	ROI drawn by radiologist manually	
Sasikala et al. [37]	-	SE-FH	Speckle reducing anisotropic diffusion	Fuzzy level set	-
Sasikala et al. [38]	Epiq 5G1/SS with Make, Philips	SE-FH	Speckle reducing anisotropic diffusion	Fuzzy level set	-
Wu et al. [39]	IU22 system, Philips; ACUSON S2000, Siemens	SE-ARF	Harris corner operation	Manually drawn from B-mode and map to elastography	-
Wu et al. [40]	IU22 system, Philips; ACUSON S2000, Siemens	SE-ARF	Fractional order operation	Manually drawn from B-mode and map to elastography	-
Yu et al. [41]	Aixplorer, SuperSonic	SWE	K-means clustering, Active contour, dyadic wavelet, transform, GAD	Manual segmentation vs. level set vs. manual editing after level set	Compared to manual segmentation using MAD, MxAD, *p* < 10×, Ao, Ad, DSC
Zhang et al. [42]	Aixplorer, SuperSonic	SWE	Image separation	Level set	-
Zhang et al. [43]	Aixplorer, SuperSonic	SWE	Image separation	RD-GAD vs. GAD	Compared with manual segmentation using TP, FP, Acc, Sp (indexed by Ao), RMSE
Zhang et al. [44] 2020	Aixplorer, SuperSonic	SWE	-	Manually segmented using an open-source image platform	DSC, ICC
Zhou et al. [45]	Aixplorer, SuperSonic	SWE	Image separation	CNN ^†^	-

^†^ Image segmentation function was not standalone and facilitated by machine learning model. Acc: Accuracy; Ao: area overlapped; Ad: area difference; ARF: acoustic radiation force; CNN: convolution neutral network; DSC: dice similarity coefficient; FH: freehand; GAD: Gabor-based anisotropic diffusion; ICC: intraclass correlation; MAD: mean absolute distance; MxAD: maximum absolute distance; NAD: normalized area difference; NCT: normalized center translation; NSM: normalized slope of metric value; *p* < 10×: percentage of points with different less than 10 pixels; PGBM: point-wise gated Boltzmann machine; RD: reaction diffusion level set; RMSE: root mean square error; Ro: radiologist; Sp: specificity; SWE: shear wave elastography; SE: strain elastography; w/: with; w/o: without.

**Table 3:** Configuration of machine learning and classification models.

Article	Data Augmentation	Transfer Learning/Pre-Training	Feature Extraction	Classification	Model Validation
Chen et al. [33]	-	-	Pre-determined image statistical features (NAD, NSM, NCT) targeted to SE characteristics	SVM	-
Fujioka et al. [34]	Classic	ImageNet	CNN (Xception, Inception V3, InceptionNesNetV2, DenseNet1, DenseNet161, NASNetMobile)		-
Misra et al. [35]	Classic	ImageNet	Ensembled (B-mode & SE) with Ensembled (AlexNet & ResNet) vs. w/o Ensembled Learning		5-fold cxv
Moon et al. [36]	-	-	Pre-set elasticity features (5 SE, 6 B-mode)	MPNN vs BC	-
Sasikala et al. [37]	-	-	Extraction: LBP vs. LTP Fusion: serial vs. parallel Reduction: PCA vs. CCA	SVM	-
Sasikala et al. [38]	-	-	GLCM vs. GLDM vs. LAW vs. LBP Fusion and Selection: PSO	SVM w/ radial bias function	10 fold cxv
Wu et al. [39]	-	-	Harris corner convolution vs. fractional order convolution, pooling	Random decision forest vs. GRNN (FCN)	0 to 10 fold cxv w/ different case ratios
Wu et al. [40]	-	-	Fractional order convolution vs. 1st Sobel w/ 2nd Laplacian order convolution,	GRNN (FCN)	0 to 10 fold cxv
Yu et al. [41]	-	-	Pre-determined textural features (26) Mutual information-based feature selection	SVM	Leave-one-out cxv
Zhang et al. [42]	-	-	GLCM vs. PGBM and RBM PCA vs. *t*-test vs. no reduction	SVM vs. KNN vs. ELM	5-fold cxv
Zhang et al. [43]	-	-	(Prime) Contourlet-based texture features (SWE) and morphological features (B-mode) vs. nextraction DPN vs. PCA vs. MKL	SVM	Leave-one-out cxv
Zhang et al. [44]	Classic	-	CNN, LASSO regression	Likelihood ratio	By external testing dataset
Zhou et al. [45]	Classic	-	CNN feature extraction w/ network forward process	CNN	By external testing dataset

BC: Bayesian classifier; CCA: canonical correlation analysis; CNN: convolution neural network; cxv: cross-validation; DPN: deep polynomial network; ELM: extreme learning machine; FCN: fully-connected network; GLCM: grey level difference matrix; GLDM: grey level difference matrix; GRNN: generalized regression neural network; KNN: K-nearest neighbour; LASSO: least absolute shrinkage and selection operator; LBP: local binary pattern; LTP: local ternary pattern; MKL: multiple kernel learning; MPNN: multilayer perceptron neural network; NAD: normalized area difference; NCT: normalized center translation; NSM: normalized slope of metric value; PCA: principal component analysis; PGBM: point-wise gated Boltzmann machine; PSO: particle swarm optimization; RBM: restricted Boltzmann machine; SE: strain elastography; SVM: support vector machine; w/: with; w/o: without.

**Table 4:** Evaluation metric and outcome performance.

Article	Remarks	Evaluation Metrics and Outcomes						
		Acc	Sn/Rc	Sp	PPV/Pc	NPV	AUC	Others
Chen et al. [33]	-	91.00%	85.00%	95.00%	91.89%	90.48%	0.936	-
Fujioka et al. [34]	Mean performance of all CNNs and Epochs * vs. radiologist readouts	-	84.3%	78.9%	-	-	0.870	-
Misra et al. [35]	w/ * vs. w/o manual cropping Ensembled * vs. w/o ensembled learning	87.48%	85.18%	89.65%	88.49%	-	-	F1 = 0.868
Moon et al. [36]	MPNN * vs. BC	-	92%	74%	58%	96%	0.89	-
Sasikala et al. [37]	LBP vs. LTP * Serial * vs. parallel PCA vs. CCA *	98.2%	96.2%	100.0%	-	-	-	F1 = 0.981 MCC = 0.965 K = 0.964 BCR = 98.08%
Sasikala et al. [38]	GLCM vs. GLDM vs. LAW vs. LBP *	96.2%	94.4%	97.4%	96.2%	-	-	F1 = 0.953 MCC = 0.921 BCR = 95.88%
Wu et al. [39]	Harris corner * vs. fractional-order Random decision forest * vs. GRNN	86.97%	86.02%	87.63%	-	-	-	F1 = 0.86
Wu et al. [40]	Fractional order * vs. 2nd order convolution	87.86%	92.92%	-	80.42%	94.22	-	F1 = 0.862
Yu et al. [41]	Manual vs. level set vs. level set + post-manual edit *	94.8%	95.1%	94.6%	91.9%	96.8%	-	YI = 89.7%
Zhang et al. [42]	Level set vs. PGBM vs. PGBM w/ RBM * PCA vs. *t*-test vs. no reduction * ELM vs. KNN vs. SVM *	93.4%	88.6%	97.1%	-	-	0.947	YI = 85.7%
Zhang et al. [43]	Contourlet * vs. raw PCA vs. MKL vs. DPN *	95.6%	97.8%	94.1%	-	-	0.961	YI = 91.9%
Zhang et al. [44]	B-mode vs. SWE * vs. BI-RADS at US External testing set result	-	100%	100%	-	-	1.00	(+)LR = ∝ (−)LR = 0
Zhou et al. [45]	11 layers vs 13 layers vs 16 layers *	95.8%	96.2%	95.7%	-	-	-	-

* indicates the model that had the results presented in this table, which was either the proposed model in the article or the best-performing model. Acc: accuracy: Sn: sensitivity; Rc: recall; Sp: specificity; PPV: positive predictive value; Pc: precision; NPV: negative predictive value; AUC: area under receiver-operating curve; MCC: Matthews correlation coefficient; BCR: balance classification rate; LR: likelihood ratio; YI: Youden’s index. BC: Bayesian classifier; BIRADS: Breast Imaging Reporting and Data System; CCA: canonical correlation analysis; CNN: convolution neural network; DPN: deep polynomial network; ELM: extreme learning machine; GLCM: gray level difference matrix; GLDM: gray level difference matrix; GRNN: generalized regression neural network; K-nearest neighbour; LBP: local binary pattern; LTP: local ternary pattern; MKL: multiple kernel learning; MPNN: multilayer perceptron neural network; PCA: principal component analysis; PGBM: point-wise gated Boltzmann machine; RBM: restricted Boltzmann machine; SE: strain elastography; SWE: shear wave elastography; SVM: support vector machine; w/: with; w/o: without.

## References

[1] Sun Y.-S., Zhao Z., Yang Z.-N., Xu F., Lu H.-J., Zhu Z.-Y., Shi W., Jiang J., Yao P.-P., Zhu H.-P. (2017). Risk Factors and Preventions of Breast Cancer. Int. J. Biol. Sci..

[2] Harbeck N., Gnant M. (2017). Breast cancer. Lancet.

[3] Benson J.R., Jatoi I. (2012). The global breast cancer burden. Future Oncol..

[4] Cao W., Chen H.-D., Yu Y.-W., Li N., Chen W.-Q. (2021). Changing profiles of cancer burden worldwide and in China: A secondary analysis of the global cancer statistics 2020. Chin. Med. J..

[5] Sung H., Ferlay J., Siegel R.L., Laversanne M., Soerjomataram I., Jemal A., Bray F. (2021). Global cancer statistics 2020: GLOBOCAN estimates of incidence and mortality worldwide for 36 cancers in 185 countries. CA Cancer J. Clin..

[6] Gross C.P., Long J.B., Ross J.S., Abu-Khalaf M.M., Wang R., Killelea B.K., Gold H.T., Chagpar A.B., Ma X. (2013). The cost of breast cancer screening in the Medicare population. JAMA Intern. Med..

[7] Lowry K.P., Trentham-Dietz A., Schechter C.B., Alagoz O., Barlow W.E., Burnside E.S., Conant E.F., Hampton J.M., Huang H., Kerlikowske K. (2019). Long-Term Outcomes and Cost-Effectiveness of Breast Cancer Screening With Digital Breast Tomosynthesis in the United States. JNCI J. Natl. Cancer Inst..

[8] Coleman M.P., Quaresma M., Berrino F., Lutz J.-M., De Angelis R., Capocaccia R., Baili P., Rachet B., Gatta G., Hakulinen T. (2008). Cancer survival in five continents: A worldwide population-based study (CONCORD). Lancet Oncol..

[9] Qaseem A., Lin J.S., Mustafa R.A., Horwitch C.A., Wilt T.J. (2019). Screening for breast cancer in average-risk women: A guidance statement from the American College of Physicians. Ann. Intern. Med..

[10] Keith L.G., Oleszczuk J.J., Laguens M. (2002). Are mammography and palpation sufficient for breast cancer screening? A dissenting opinion. J. Women’s Health Gend.-Based Med..

[11] Pauwels E.K.J., Foray N., Bourguignon M.H. (2016). Breast Cancer Induced by X-Ray Mammography Screening? A Review Based on Recent Understanding of Low-Dose Radiobiology. Med. Princ. Pract..

[12] Seely J., Alhassan T. (2018). Screening for breast cancer in 2018—what should we be doing today?. Curr. Oncol..

[13] Lehman C.D., DeMartini W., Anderson B.O., Edge S.B., Robinson K.G. (2009). Indications for Breast MRI in the Patient with Newly Diagnosed Breast Cancer. J. Natl. Compr. Cancer Netw..

[14] Lehman C.D., Gatsonis C., Kuhl C.K., Hendrick R.E., Pisano E.D., Hanna L., Peacock S., Smazal S.F., Maki D.D., Julian T.B. (2007). MRI Evaluation of the Contralateral Breast in Women with Recently Diagnosed Breast Cancer. N. Engl. J. Med..

[15] Morris E.A. (2002). Breast cancer imaging with MRI. Radiol. Clin..

[16] Da Costa Vieira R.A., Biller G., Uemura G., Ruiz C.A., Curado M.P. (2017). Breast cancer screening in developing countries. Clinics.

[17] Teh W., Wilson A.R.M. (1998). The role of ultrasound in breast cancer screening. A consensus statement by the European Group for breast cancer screening. Eur. J. Cancer.

[18] McKnight A.L., Kugel J.L., Rossman P.J., Manduca A., Hartmann L.C., Ehman R.L. (2002). MR Elastography of Breast Cancer: Preliminary Results. Am. J. Roentgenol..

[19] Ophir J., Cespedes I., Ponnekanti H., Yazdi Y., Li X. (1991). Elastography: A quantitative method for imaging the elasticity of biological tissues. Ultrason. Imaging.

[20] Sohn Y.-M., Kim M.J., Kim E.-K., Kwak J.Y., Moon H.J., Kim S.J. (2009). Sonographic elastography combined with conventional sonography: How much is it helpful for diagnostic performance?. J. Ultrasound Med..

[21] Itoh A., Ueno E., Tohno E., Kamma H., Takahashi H., Shiina T., Yamakawa M., Matsumura T. (2006). Breast disease: Clinical application of US elastography for diagnosis. Radiology.

[22] Bercoff J., Tanter M., Fink M. (2004). Supersonic shear imaging: A new technique for soft tissue elasticity mapping. IEEE Trans. Ultrason. Ferroelectr. Freq. Control.

[23] Yamakoshi Y., Nakajima T., Kasahara T., Yamazaki M., Koda R., Sunaguchi N. (2016). Shear wave imaging of breast tissue by color Doppler shear wave elastography. IEEE Trans. Ultrason. Ferroelectr. Freq. Control.

[24] Fu L.-n., Yi W., Yong W., Huang Y.-h. (2011). Value of ultrasound elastography in detecting small breast tumors. Chin. Med. J..

[25] Faruk T., Islam M.K., Arefin S., Haq M.Z. (2015). The Journey of Elastography: Background, Current Status, and Future Possibilities in Breast Cancer Diagnosis. Clin. Breast Cancer.

[26] Jiang W.-w., Li A.-h., Zheng Y.-P. (2014). A semi-automated 3-D annotation method for breast ultrasound imaging: System development and feasibility study on phantoms. Ultrasound Med. Biol..

[27] Samir A.E., Dhyani M., Vij A., Bhan A.K., Halpern E.F., Méndez-Navarro J., Corey K.E., Chung R.T. (2015). Shear-Wave Elastography for the Estimation of Liver Fibrosis in Chronic Liver Disease: Determining Accuracy and Ideal Site for Measurement. Radiology.

[28] Kerridge W.D., Kryvenko O.N., Thompson A., Shah B.A. (2015). Fat Necrosis of the Breast: A Pictorial Review of the Mammographic, Ultrasound, CT, and MRI Findings with Histopathologic Correlation. Radiol. Res. Pract..

[29] Fernandes Chala L., de Barros N., de Camargo Moraes P., Endo É., Kim S.J., Maciel Pincerato K., Carvalho F.M., Guido Cerri G. (2004). Fat necrosis of the breast: Mammographic, sonographic, computed tomography, and magnetic resonance imaging findings. Curr. Probl. Diagn. Radiol..

[30] Yassin N.I.R., Omran S., El Houby E.M.F., Allam H. (2018). Machine learning techniques for breast cancer computer aided diagnosis using different image modalities: A systematic review. Comput. Methods Programs Biomed..

[31] Sawyer Lee R., Dunnmon J.A., He A., Tang S., Ré C., Rubin D.L. (2021). Comparison of segmentation-free and segmentation-dependent computer-aided diagnosis of breast masses on a public mammography dataset. J. Biomed. Inform..

[32] Tricco A.C., Lillie E., Zarin W., O’Brien K.K., Colquhoun H., Levac D., Moher D., Peters M.D., Horsley T., Weeks L. (2018). PRISMA extension for scoping reviews (PRISMA-ScR): Checklist and explanation. Ann. Intern. Med..

[33] Chen C.J., Chang R.F., Moon W.K., Chen D.R., Wu H.K. (2006). 2-D ultrasound strain images for breast cancer diagnosis using nonrigid subregion registration. Ultrasound Med. Biol..

[34] Fujioka T., Katsuta L., Kubota K., Mori M., Kikuchi Y., Kato A., Oda G., Nakagawa T., Kitazume Y., Tateishi U. (2020). Classification of Breast Masses on Ultrasound Shear Wave Elastography using Convolutional Neural Networks. Ultrason. Imaging.

[35] Misra S., Jeon S., Managuli R., Lee S., Kim G., Yoon C., Lee S., Barr R.G., Kim C. (2021). Bi-modal Transfer Learning for Classifying Breast Cancers via Combined B-mode and Ultrasound Strain Imaging. IEEE Trans. Ultrason. Ferroelectr. Freq. Control.

[36] Moon W.K., Choi J.W., Cho N., Park S.H., Chang J.M., Jang M., Kim K.G. (2010). Computer-aided analysis of ultrasound elasticity images for classification of benign and malignant breast masses. AJR Am. J. Roentgenol.

[37] Sasikala S., Ezhilarasi M., Senthil S. (2018). Breast Cancer Diagnosis System Based on the Fusion of Local Binary and Ternary Patterns from Ultrasound B Mode and Elastography Images. Curr. Med. Imaging.

[38] Sasikala S., Bharathi M., Ezhilarasi M., Senthil S., Reddy M.R. (2019). Particle swarm optimization based fusion of ultrasound echographic and elastographic texture features for improved breast cancer detection. Australas. Phys. Eng. Sci. Med..

[39] Wu J.X., Chen P.Y., Lin C.H., Chen S.G., Shung K.K. (2020). Breast Benign and Malignant Tumors Rapidly Screening by ARFI-VTI Elastography and Random Decision Forests Based Classifier. IEEE Access.

[40] Wu J.X., Liu H.C., Chen P.Y., Lin C.H., Chou Y.H., Shung K.K. (2020). Enhancement of ARFI-VTI Elastography Images in Order to Preliminary Rapid Screening of Benign and Malignant Breast Tumors Using Multilayer Fractional-Order Machine Vision Classifier. IEEE Access.

[41] Yu Y.Y., Xiao Y., Cheng J.Y., Chiu B. (2018). Breast lesion classification based on supersonic shear-wave elastography and automated lesion segmentation from B-mode ultrasound images. Comput. Biol. Med..

[42] Zhang Q., Xiao Y., Dai W., Suo J.F., Wang C.Z., Shi J., Zheng H.R. (2016). Deep learning based classification of breast tumors with shear-wave elastography. Ultrasonics.

[43] Zhang Q., Song S., Xiao Y., Chen S., Shi J., Zheng H.R. (2019). Dual-mode artificially-intelligent diagnosis of breast tumours in shear-wave elastography and B-mode ultrasound using deep polynomial networks. Med. Eng. Phys..

[44] Zhang X., Liang M., Yang Z.H., Zheng C.S., Wu J.Y., Ou B., Li H.J., Wu X.Y., Luo B.M., Shen J. (2020). Deep Learning-Based Radiomics of B-Mode Ultrasonography and Shear-Wave Elastography: Improved Performance in Breast Mass Classification. Front. Oncol..

[45] Zhou Y.J., Xu J.X., Liu Q.G., Li C., Liu Z.Y., Wang M.Y., Zheng H.R., Wang S.S. (2018). A Radiomics Approach with CNN for Shear-Wave Elastography Breast Tumor Classification. IEEE Trans. Biomed. Eng..

[46] Refaeilzadeh P., Tang L., Liu H. (2009). Cross-validation. Encycl. Database Syst..

[47] Yuen Q.W.-H., Zheng Y.-P., Huang Y.-P., He J.-F., Cheung J.C.-W., Ying M. (2011). In-vitro strain and modulus measurements in porcine cervical lymph nodes. Open Biomed. Eng. J..

[48] Hoskins P.R., Hoskins P.R., Martin K., Thrush A. (2019). Elastography. Diagnositic Ultrasound: Physics and Equipment.

[49] Regner D.M., Hesley G.K., Hangiandreou N.J., Morton M.J., Nordland M.R., Meixner D.D., Hall T.J., Farrell M.A., Mandrekar J.N., Harmsen W.S. (2006). Breast lesions: Evaluation with US strain imaging–clinical experience of multiple observers. Radiology.

[50] Barr R.G., De Silvestri A., Scotti V., Manzoni F., Rebuffi C., Capittini C., Tinelli C. (2019). Diagnostic performance and accuracy of the 3 interpreting methods of breast strain elastography: A systematic review and meta-analysis. J. Ultrasound Med..

[51] Barr R.G. (2018). The role of sonoelastography in breast lesions. Semin. Ultrasound CT MRI.

[52] Ying M., Zheng Y.-P., Kot B.C.-W., Cheung J.C.-W., Cheng S.C.-H., Kwong D.L.-W. (2013). Three-dimensional elastography for cervical lymph node volume measurements: A study to investigate feasibility, accuracy and reliability. Ultrasound Med. Biol..

[53] Sarvazyan A.P., Rudenko O.V., Swanson S.D., Fowlkes J.B., Emelianov S.Y. (1998). Shear wave elasticity imaging: A new ultrasonic technology of medical diagnostics. Ultrasound Med. Biol..

[54] Berg W.A., Cosgrove D.O., Doré C.J., Schäfer F.K., Svensson W.E., Hooley R.J., Ohlinger R., Mendelson E.B., Balu-Maestro C., Locatelli M. (2012). Shear-wave elastography improves the specificity of breast US: The BE1 multinational study of 939 masses. Radiology.

[55] Lee S.H., Chang J.M., Kim W.H., Bae M.S., Seo M., Koo H.R., Chu A.J., Gweon H.M., Cho N., Moon W.K. (2014). Added value of shear-wave elastography for evaluation of breast masses detected with screening US imaging. Radiology.

[56] Evans A., Whelehan P., Thomson K., McLean D., Brauer K., Purdie C., Jordan L., Baker L., Thompson A. (2010). Quantitative shear wave ultrasound elastography: Initial experience in solid breast masses. Breast Cancer Res..

[57] Xiao Y., Zeng J., Niu L., Zeng Q., Wu T., Wang C., Zheng R., Zheng H. (2014). Computer-aided diagnosis based on quantitative elastographic features with supersonic shear wave imaging. Ultrasound Med. Biol..

[58] Zhang Q., Xiao Y., Chen S., Wang C., Zheng H. (2015). Quantification of elastic heterogeneity using contourlet-based texture analysis in shear-wave elastography for breast tumor classification. Ultrasound Med. Biol..

[59] Pu Y.-F., Zhou J.-L., Yuan X. (2009). Fractional differential mask: A fractional differential-based approach for multiscale texture enhancement. IEEE Trans. Image Process..

[60] Liu Y., Liu S., Cao Y., Wang Z. (2015). Automatic chessboard corner detection method. IET Image Process..

[61] Yu Y., Acton S.T. (2002). Speckle reducing anisotropic diffusion. IEEE Trans. Image Process..

[62] Zou K.H., Warfield S.K., Bharatha A., Tempany C.M., Kaus M.R., Haker S.J., Wells III W.M., Jolesz F.A., Kikinis R. (2004). Statistical validation of image segmentation quality based on a spatial overlap index1: Scientific reports. Acad. Radiol..

[63] O’Mahony N., Campbell S., Carvalho A., Harapanahalli S., Hernandez G.V., Krpalkova L., Riordan D., Walsh J. Deep learning vs. traditional computer vision. Proceedings of the Science and Information Conference.

[64] Xue H., Yang Q., Chen S. (2009). SVM: Support vector machines. The Top Ten Algorithms in Data Mining.

[65] Dumoulin V., Visin F. (2016). A guide to convolution arithmetic for deep learning. arXiv.

[66] Horiguchi S., Ikami D., Aizawa K. (2019). Significance of softmax-based features in comparison to distance metric learning-based features. IEEE Trans. Pattern Anal. Mach. Intell..

[67] Deng J., Dong W., Socher R., Li L.-J., Li K., Fei-Fei L. Imagenet: A large-scale hierarchical image database. Proceedings of the 2009 IEEE Conference on Computer Vision and Pattern Recognition.

[68] Morid M.A., Borjali A., Del Fiol G. (2021). A scoping review of transfer learning research on medical image analysis using ImageNet. Comput. Biol. Med..

[69] Cheplygina V., de Bruijne M., Pluim J.P. (2019). Not-so-supervised: A survey of semi-supervised, multi-instance, and transfer learning in medical image analysis. Med. Image Anal..

[70] Szegedy C., Liu W., Jia Y., Sermanet P., Reed S., Anguelov D., Erhan D., Vanhoucke V., Rabinovich A. Going deeper with convolutions. Proceedings of the IEEE Conference on Computer Vision and Pattern Recognition.

[71] Szegedy C., Vanhoucke V., Ioffe S., Shlens J., Wojna Z. Rethinking the inception architecture for computer vision. Proceedings of the IEEE Conference on Computer Vision and Pattern Recognition.

[72] Szegedy C., Ioffe S., Vanhoucke V., Alemi A.A. Inception-v4, inception-resnet and the impact of residual connections on learning. Proceedings of the Thirty-first AAAI Conference on Artificial Intelligence.

[73] Zoph B., Vasudevan V., Shlens J., Le Q.V. Learning transferable architectures for scalable image recognition. Proceedings of the IEEE Conference on Computer Vision and Pattern Recognition.

[74] Huang G., Liu Z., Van Der Maaten L., Weinberger K.Q. Densely connected convolutional networks. Proceedings of the IEEE Conference on Computer Vision and Pattern Recognition.

[75] Krizhevsky A., Sutskever I., Hinton G.E. (2012). Imagenet classification with deep convolutional neural networks. Adv. Neural Inf. Process. Syst..

[76] He K., Zhang X., Ren S., Sun J. Deep residual learning for image recognition. Proceedings of the IEEE Conference on Computer Vision and Pattern Recognition.

[77] Chicco D., Jurman G. (2020). The advantages of the Matthews correlation coefficient (MCC) over F1 score and accuracy in binary classification evaluation. BMC Genom..

[78] Matthews B.W. (1975). Comparison of the predicted and observed secondary structure of T4 phage lysozyme. Biochim. Biophys. Acta Protein Struct..

[79] Chicco D. (2017). Ten quick tips for machine learning in computational biology. BioData Min..

[80] Fawcett T. (2006). An introduction to ROC analysis. Pattern Recognit. Lett..

[81] Colquhoun D. (2014). An investigation of the false discovery rate and the misinterpretation of *p*-values. R. Soc. Open Sci..

[82] Xie J., Liu R., Luttrell IV J., Zhang C. (2019). Deep learning based analysis of histopathological images of breast cancer. Front. Genet..

[83] Bowers A.J., Zhou X. (2019). Receiver operating characteristic (ROC) area under the curve (AUC): A diagnostic measure for evaluating the accuracy of predictors of education outcomes. J. Educ. Stud. Placed Risk.

[84] Shankar V., Roelofs R., Mania H., Fang A., Recht B., Schmidt L. Evaluating machine accuracy on imagenet. Proceedings of the International Conference on Machine Learning.

[85] Santos M.S., Soares J.P., Abreu P.H., Araujo H., Santos J. (2018). Cross-validation for imbalanced datasets: Avoiding overoptimistic and overfitting approaches [research frontier]. IEEE Comput. Intell. Mag..

[86] Yusuf M., Atal I., Li J., Smith P., Ravaud P., Fergie M., Callaghan M., Selfe J. (2020). Reporting quality of studies using machine learning models for medical diagnosis: A systematic review. BMJ Open.

[87] Probst P., Boulesteix A.-L., Bischl B. (2019). Tunability: Importance of hyperparameters of machine learning algorithms. J. Mach. Learn. Res..

[88] Russell S., Norvig P. (2002). Artificial Intelligence: A Modern Approach.

[89] Gareth J., Daniela W., Trevor H., Robert T. (2013). An introduction to Statistical Learning: With Applications in R.

[90] Ripley B.D. (2007). Pattern Recognition and Neural Networks.

[91] Kuhn M., Johnson K. (2013). Applied Predictive Modeling.

[92] Gee A.H., Housden R.J., Hassenpflug P., Treece G.M., Prager R.W. (2006). Sensorless freehand 3D ultrasound in real tissue: Speckle decorrelation without fully developed speckle. Med. Image Anal..

[93] Russakovsky O., Deng J., Su H., Krause J., Satheesh S., Ma S., Huang Z., Karpathy A., Khosla A., Bernstein M. (2015). Imagenet large scale visual recognition challenge. Int. J. Comput. Vis..

[94] Roelofs R., Fridovich-Keil S., Miller J., Shankar V., Hardt M., Recht B., Schmidt L. A meta-analysis of overfitting in machine learning. Proceedings of the 33rd International Conference on Neural Information Processing Systems.

[95] Cabitza F., Rasoini R., Gensini G.F. (2017). Unintended consequences of machine learning in medicine. JAMA.

[96] Geiger R.S., Cope D., Ip J., Lotosh M., Shah A., Weng J., Tang R. (2021). “Garbage in, garbage out” revisited: What do machine learning application papers report about human-labeled training data?. Quant. Sci. Stud..

[97] Vaswani A., Shazeer N., Parmar N., Uszkoreit J., Jones L., Gomez A.N., Kaiser Ł., Polosukhin I. Attention is all you need. Proceedings of the Advances in Neural Information Processing Systems.

[98] Ronneberger O., Fischer P., Brox T. U-net: Convolutional networks for biomedical image segmentation. Proceedings of the International Conference on Medical Image Computing and Computer-Assisted Intervention.

[99] Lee C.S., Lee A.Y. (2020). Clinical applications of continual learning machine learning. Lancet Digit. Health.

